# Small methyltransferase RlmH assembles a composite active site to methylate a ribosomal pseudouridine

**DOI:** 10.1038/s41598-017-01186-5

**Published:** 2017-04-20

**Authors:** Cha San Koh, Rohini Madireddy, Timothy J. Beane, Phillip D. Zamore, Andrei A. Korostelev

**Affiliations:** 1 RNA Therapeutics Institute, University of Massachusetts Medical School, 368 Plantation St, Worcester, MA 01605 USA; 2 RNA Therapeutics Institute, Howard Hughes Medical Institute, University of Massachusetts Medical School, 368 Plantation St, Worcester, MA 01605 USA

## Abstract

Eubacterial ribosomal large-subunit methyltransferase H (RlmH) methylates 23S ribosomal RNA pseudouridine 1915 (Ψ1915), which lies near the ribosomal decoding center. The smallest member of the SPOUT superfamily of methyltransferases, RlmH lacks the RNA recognition domain found in larger methyltransferases. The catalytic mechanism of RlmH enzyme is unknown. Here, we describe the structures of RlmH bound to S-adenosyl-methionine (SAM) and the methyltransferase inhibitor sinefungin. Our structural and biochemical studies reveal catalytically essential residues in the dimer-mediated asymmetrical active site. One monomer provides the SAM-binding site, whereas the conserved C-terminal tail of the second monomer provides residues essential for catalysis. Our findings elucidate the mechanism by which a small protein dimer assembles a functionally asymmetric architecture.

## Introduction

Post-transcriptional, covalent modifications of nucleobases and ribose sugars modulate RNA function^1–3^. Ribosomal RNA (rRNA) is modified primarily near key functional sites, including the decoding center responsible for accurate translation of messenger RNA into protein^4, 5^. In bacteria, uridine 1915 of the 23S rRNA undergoes two steps of post-transcriptional modification—pseudouridylation, followed by N3-methylation—that convert it to 3-methylpseudouridine (m^3^Ψ). Uridine 1915 lies in the hairpin loop of the universally conserved helix 69. This essential^6–9^ hairpin loop docks to the decoding center of the small ribosomal subunit^10–12^ and interacts with tRNA^13–18^ and protein factors throughout translation elongation^19, 20^, termination^7, 14, 18, 21–26^ and ribosome recycling^25, 27^. Conversion of U1915 to m^3^Ψ is widely conserved among eubacteria^28^.

The ribosomal large-subunit methyltransferase H (RlmH), also known as YbeA^29–31^, catalyzes the site-specific transfer of a methyl group from S-adenosyl-methionine (SAM) to the N3 position of Ψ1915. The methyltransferase confers fitness advantage under stress conditions, as suggested by enhanced ethanol tolerance of RlmH-overexpressing cells^32^ and cell fitness in comparison with an RlmH deletion mutant^31^. RlmH does not methylate either of the neighboring (positions 1911 and 1917) or other pseudouridines in rRNA^30, 31^. RlmH acts on the assembled 70S ribosome^33^. RlmH is the smallest member of the SPOUT methyltransferases, a superfamily of enzymes that includes the tRNA-methylating enzymes SpoU (TrmH) and TrmD^28, 34–37^. Unlike most SPOUT proteins, which contain two domains—a core “knotted” domain and an RNA-binding domain^28, 38, 39^—RlmH comprises a single knotted domain. How the single RlmH domain catalyzes methyl transfer with high efficiency and site specificity is unknown.

To gain insight into the mechanism of RlmH, we determined 2.3 and 2.1 Å X-ray structures of *E. coli* RlmH with the cofactor SAM and with the SAM-mimicking inhibitor sinefungin. Sinefungin contains an amino group in place of the methyl group of SAM and inhibits SAM-dependent methylation of nucleic acids, proteins and other molecules, resulting in antifungal^40, 41^, antiviral^42, 43^ and antiprotozoal^44^ activities. Informed by the structural rearrangements in RlmH upon binding of these cofactors, we performed biochemical assays to identify residues critical for methylation of Ψ1915 in the *E. coli* 70S ribosome. Our data show that dimerization generates the RlmH active site that is composed of conserved residues from the knotted core of one monomer and from the C-terminal tail of the other. Thus, assembly of an asymmetric RlmH dimer forms a composite active site. Structural comparison of RlmH with other SPOUT proteins suggests that common structural elements in an overhand knot determine the catalytic activity of dimeric SPOUT methyltransferases.

## Results

### Three bights in an overhand-knot define the RlmH SAM-binding pocket

**Figure 1:**
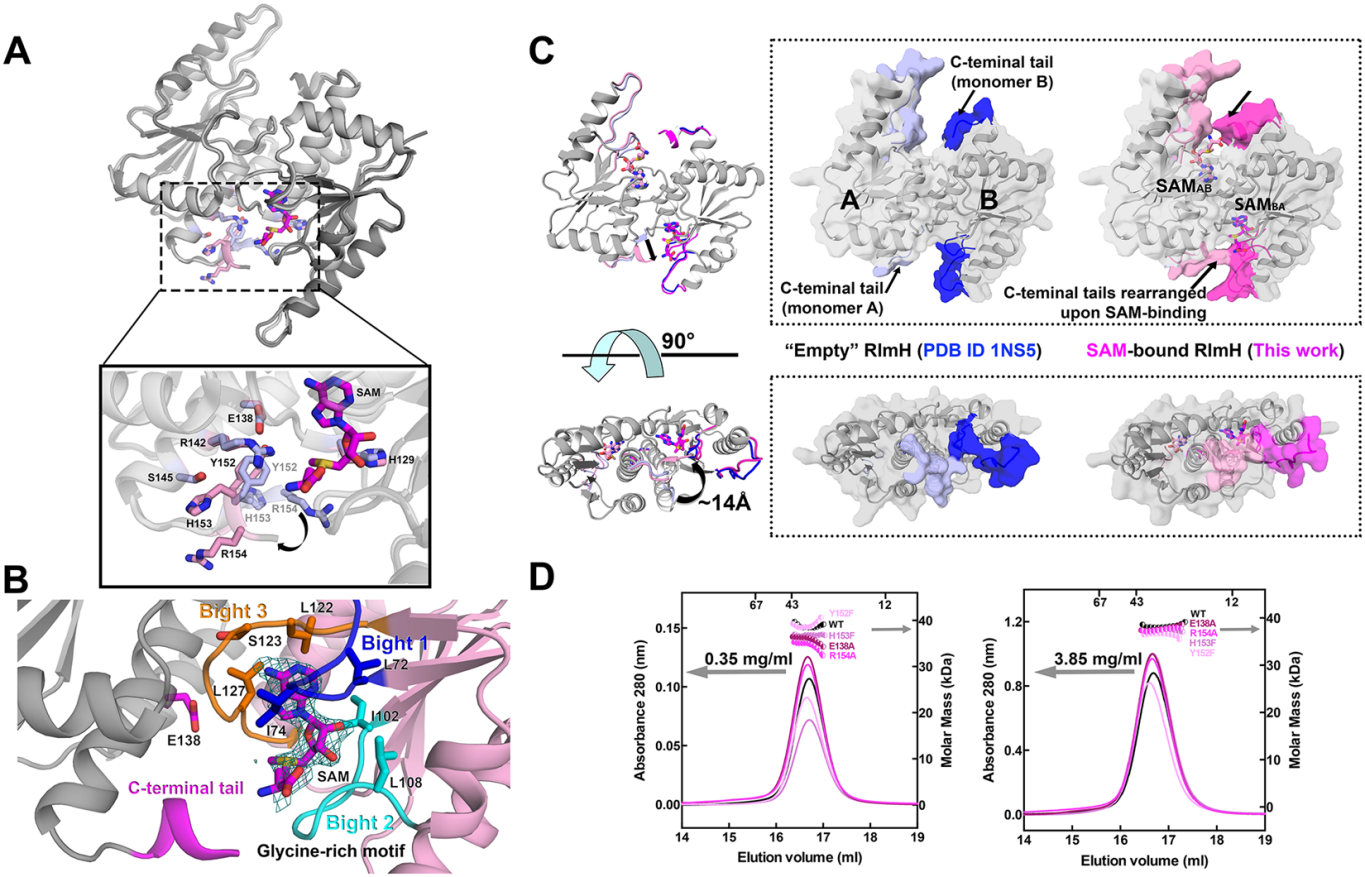
Structure and dimer formation of RlmH. **(A)** Crystal structure of the RlmH · SAM homodimer (upper panel) and close-up view of the active site (lower panel). The apo protein (light gray, PDB ID 1NS5) is superimposed on the SAM-bound protein (dark gray). Sticks show active site residues (apo: light blue; SAM-bound: pink. (**B**) Three bights (bight 1: blue; bight 2: cyan; bight 3: orange) form the SAM-binding pocket of monomer A. Teal: unbiased F_obs_-F_calc_ electron density; pink: monomer A; gray: monomer B; magenta: conserved C-terminal tail of monomer B. (**C**) Comparison of the conformations of the C-terminus in the apo- (blue) and SAM-bound protein (magenta and pink). (**D**) Molecular weights of RlmH and four mutants used in this study were determined by SEC/MALS (RI/UV). UV traces of proteins eluted from Superdex S-200 are shown. For clarity, only every tenth measurement of molar mass within the range of the elution volume is plotted. Two plots with different concentration of injected samples are shown. Gray arrows indicate the axes to which the graphs correspond. See also Figures S1, S2, S3 and S9 and Tables S1 and S2.

The crystal structures of *E. coli* RlmH bound to the cofactor SAM or to the inhibitor sinefungin contain two dimers of RlmH in an asymmetric unit, with two cofactors bound to each RlmH dimer. RlmH consists of five parallel β-strands (β2-β1-β4-β3-β5) sandwiched between two layers of α-helices: α2 and α4 on the solvent side, and α1, α5 and α3 on the dimerization interface (Figs 1, S1 and S2). This architecture comprises the overhand knot, which is the signature of the αβ-fold of SPOUT methyltransferases^28, 45^.

Each of the two cofactors (SAM and sinefungin) binds to RlmH in a bent conformation in which the amino-acid moiety of the cofactor folds toward the adenine base (Figs 1 and S3). This conformation resembles that in other ligand-bound structures of SPOUT family members^39, 46^. Because the two cofactors adopt similar conformations, we focus our discussion on the structure bound to SAM.

Three bights (U-shaped, curved sections) of the overhand knot define the SAM-binding pocket (Figs 1, S4 and S5). Bight 1 (loop β3–α3) and bight 3 (loop β5–α5) shape the hydrophobic core, which hosts the adenine moiety of SAM. Interactions with residues L72, I74, L122, L127, V132 and the backbone carbonyls of L125 and S123 secure the adenine base. The ribose of SAM interacts mainly with bight 2 (β4–α4; Figure S1). The ribose ring packs against hydrophobic side chains L102 and L108 and makes a hydrogen bond with the backbone carbonyl of L72. The homocysteine moiety of SAM is near the protein backbone of the glycine-rich motif (_103_GGPEG_107_) and D73 (Figs 1 and S3). The reactive methyl group in SAM is sandwiched between the backbone atoms of bights 2 and 3. The methyl group faces the C-terminus of the partner RlmH monomer, where the RNA substrate likely binds. This putative RNA binding site presents positively charged residues from both monomer A (K11, K42 and R43) and monomer B (R25, R26, R142 and R154), which likely contribute to binding of helix 69. The conserved electropositive patch resembles the tRNA-binding site of TrmD^47^. In TrmD, positively charged residues bind the anticodon stem loop of tRNA, with K162 - positioned similarly to R154 of RlmH - stabilizing the backbone of adenosine 38, whose neighbor guanosine 37 flips out for modification.

The SAM-binding bights 1 and 3 from one monomer interact with the same bights of the second monomer *via* hydrophobic interactions (I74, L122, L125 and L127) (Figure S1). This hydrophobic patch is conserved among RlmH orthologs (Fig. 1). The head-to-tail dimerization of RlmH contrasts the tail-to-tail dimerization of archaeal and eukaryotic counterparts responsible for m^1^Ψ or m^1^acp^3^Ψ methylation^48–50^, which resembles the bacterial SpoU sub-family of SPOUT methyltransferases (Figures S5 and S6). Yet all SPOUT family members retain the over-hand-knot architecture comprising three bights forming the SAM binding pocket.

### Cofactor binding rearranges the RlmH C-terminus

Both cofactor-bound (this work) and ligand-free (PDB ID 1NS5) RlmH form dimers. Dimerization buries ~13% (~1,100 Å^2^)^51^ of the total surface area of each monomer. In the RlmH · SAM dimer, the SAM-binding site of monomer A lies next to the C-terminus of monomer B, forming the putative helix 69-binding site. Comparing the structure of SAM-bound and ligand-free enzyme (Fig. 1) shows that cofactor binding rearranges the C-terminal loop into a 3_10_-helix in each subunit (Figs 1 and S3). Formation of the 3_10_-helix was not observed in the recent structures of SAM-bound OrfX, the *Staphylococcus aureus* homolog of RlmH^45^. The OrfX · SAM complex contains a disordered C-terminal loop and a phosphate ion bound next to the methyl group of SAM. The phosphate ion has been proposed to occupy the putative position of the phosphate backbone of the substrate rRNA nucleotide^45^, suggesting that the C-terminal tail may undergo an additional rearrangement when RlmH binds the ribosome. Thus, the C-terminal tail appears to play a role in the catalytic mechanism of RlmH, pre-arranging the C-terminal residues for RNA binding or positioning the SAM methyl group toward the RNA binding pocket, as observed for RlmH · SAM in this study (Fig. 1).

### E138 and all C-terminal mutants are catalytically inactive

The catalytic residues of RlmH have not yet been identified^45^. Alignment of 5,000 non-redundant sequences of RlmH homologs reveals that the SAM-binding pocket residue H129, residues E138 and R142 near the methyl group of SAM and the C-terminal residues Y152, H153 and R154, as evinced in the crystal structure of RlmH · SAM, are widely conserved (Figure S4). To test the functional importance of these amino acids, we generated mutant proteins and measured their catalytic activities. In particular, we sought to test whether active site pre-assembly requires Y152, H153 and R154 in the C-terminus and whether residues H129, E138 and R142 are critical for catalysis.

We purified seven single-amino-acid mutant RlmH proteins: H129A, E138A, E138Q, R142A, Y152F, H153F and R154A. The isosteric E138Q mutant was designed to test the importance of the carboxylate anion of E138 in catalyzing methyl transfer. Y152F tests the role of the hydroxyl group, which interacts with the carboxyl groups of E138 and the guanidinium of R142, in organizing the active site. H153F tests the role of the histidine hydrogen bond with S145, which connects the 3_10_-helix with α5 in the SAM-bound enzyme (Fig. 1).

**Figure 2:**
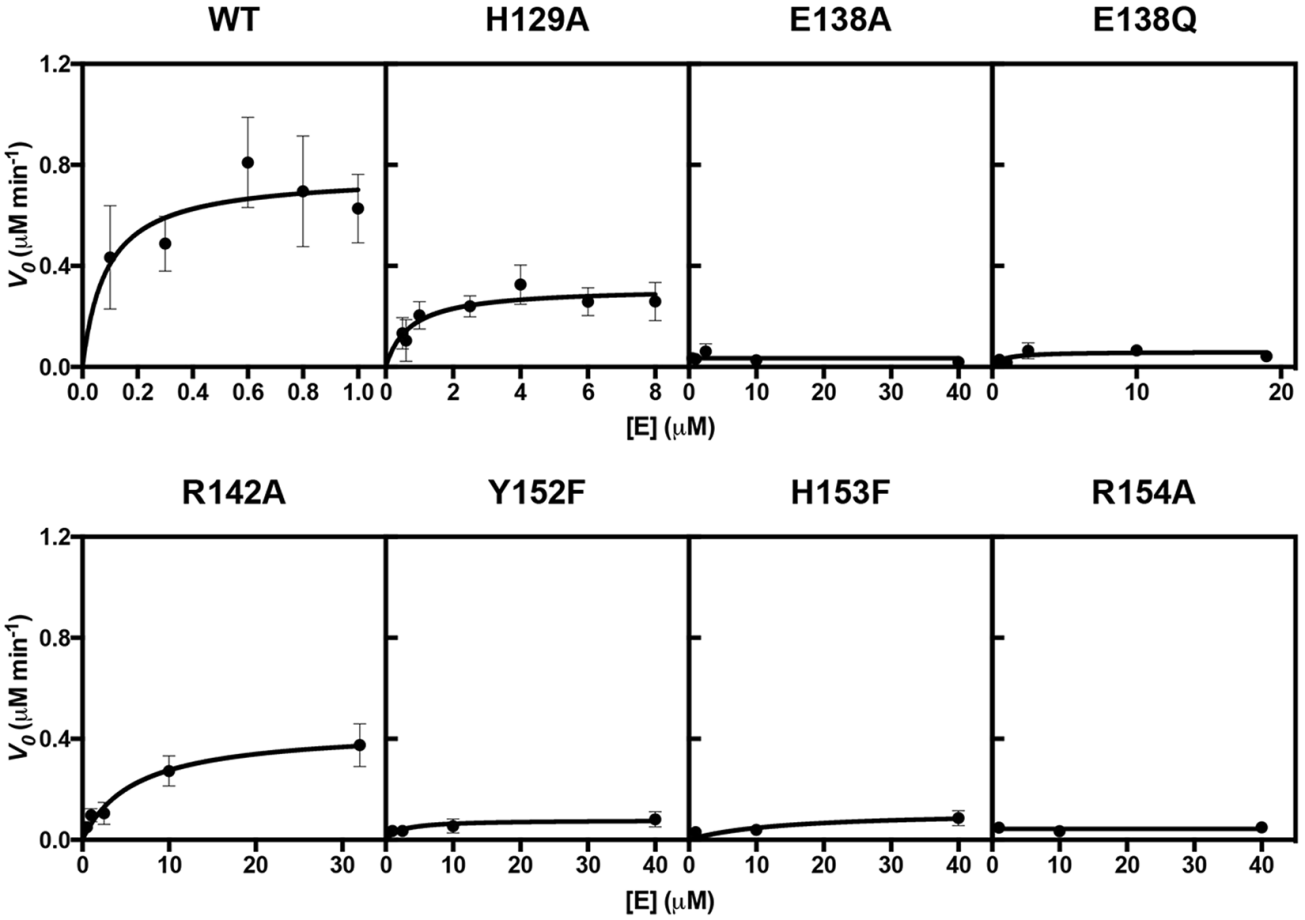
Kinetic analyses of wild-type and mutant RlmH. Initial velocities (*V*_0_, µM min^−1^) of methylation of Δ*rlmH*−70S ribosome (0.3 µM) by wild-type or mutant RlmH at 25 °C with 50 µM [^3^H]-labeled SAM. Error bars show error of fit for two independent experiments.

The activity of recombinant RlmH was tested by measuring the methylation of purified *E. coli* 70S ribosomes isolated from Δ*rlmH* cells^52^, whose 23S rRNA contains unmethylated Ψ1915. For wild-type RlmH, we measured an apparent *K*_*M*_ for substrate 70S ribosomes ≤0.3 µM; *k*_*cat*_ was 2.5 ± 0.4 min^−1^ (Fig. 2 and Table 1). These values, measured using N-terminal His-tagged RlmH, agree well with reported values for untagged RlmH^33^ (*K*_*M*_ for 70S = 0.51 ± 0.06 µM, *k*_*cat*_ = 4.95 ± 1.10 min^−1^). The catalytic activity of RlmH in our study (*k*_*cat*_/*K*_*M*_ ≥ 8.3 min^−1^ · µM^−1^) is therefore similar to that of native RlmH (*k*_*cat*_/*K*_*M*_ = 9.71 min^−1^ · µM^−1^)^33^.

**Table 1:** Michaelis-Menten analyses of methylation of Δ*rlmH*-70S ribosomes by RlmH.

	K_M_ (μM)	Fold Change K_M MUT_/K_M WT_	V_max_ (μM · min^−1^)	[E_T_] (μM)	k_cat_ (min^−1^)	Fold Change k_cat MUT_/k_cat WT_	k_cat_/K_M_ (μM^−1^ · min^−1^)	Fold Change k_cat_/K_M_
WT	≤0.3	1.0	(7.6 ± 1.1) × 10^−1^	0.3	2.5 ± 0.4	1.0	≥8.3 ± 1.3	1.00
H129A	0.7 ± 0.3	2.3	(3.1 ± 0.3) × 10^−1^	0.3	1.0 ± 0.1	0.4	1.4 ± 0.6	≤0.17
E138A	—	—	—	—	—	—	—	—
E138Q	—	—	—	—	—	—	—	—
R142A	6 ± 2	20.0	(4.4 ± 0.4) × 10^−1^	0.3	1.5 ± 0.1	0.6	(2.5 ± 0.8) × 10^−1^	≤0.03
Y152F	—	—	—	—	—	—	—	—
H153F	—	—	—	—	—	—	—	—
R154A	—	—	—	—	^—^	—	—	—

Fitted Michaelis-Menten curves are shown in Fig. 2. Values for catalytically inactive mutants E138A, E138Q, H153F and R154A are not reported. The table reports mean ± fitting error for two replicates.

None of the three C-terminal or E138 mutants (E138A, E138Q) mutants retained detectable catalytic activity. The failure of glutamine 138 to catalyze methyl transfer suggests that RlmH activity requires the acidic carboxyl group of the native glutamic acid.

Replacement of H129 or R142 with alanine reduced methyltransferase activity. H129A retained ~50% of the wild-type methylation activity, while R142A mutant protein had a *K*_*M*_ for ribosomes ≥20 times greater and a *k*_*cat*_/*K*_*M*_ ≥ 30-fold lower than wild-type RlmH (Table 1).

### Wild-type and all mutant RlmH form dimers

Loss of catalytic activity in the E138, Y152, H153 and R154 mutants might reflect (1) a failure to form dimers; (2) loss of substrate(s) binding; or (3) inactivation of the active site. We sought to distinguish among these explanations. First, we measured the oligomeric state of purified wild-type and mutant RlmH in solution using size-exclusion chromatography combined with multi-angle light scattering (SEC/MALS^53, 54^). The protein was dialyzed to remove endogenous ligand(s) that may co-purify with RlmH. SEC/MALS measures molecular mass irrespective of the shape of a protein or complex. At both ~20 and ~200 µM, each of the five proteins (wild-type, E138, Y152, H153, and R154) existed as a single peak of monodisperse particles with a molecular weight corresponding to a dimer (Fig. 1 and Table S1). Conventional size exclusion chromatography suggests that the two other mutants, H129A and R142A, similarly retain the ability to dimerize, because the two mutants had elution profiles identical to that of wild-type RlmH (data not shown). We conclude that all the mutants analyzed here retain the dimeric state of wild-type RlmH.

### RlmH mutants retain SAM binding

**Figure 3:**
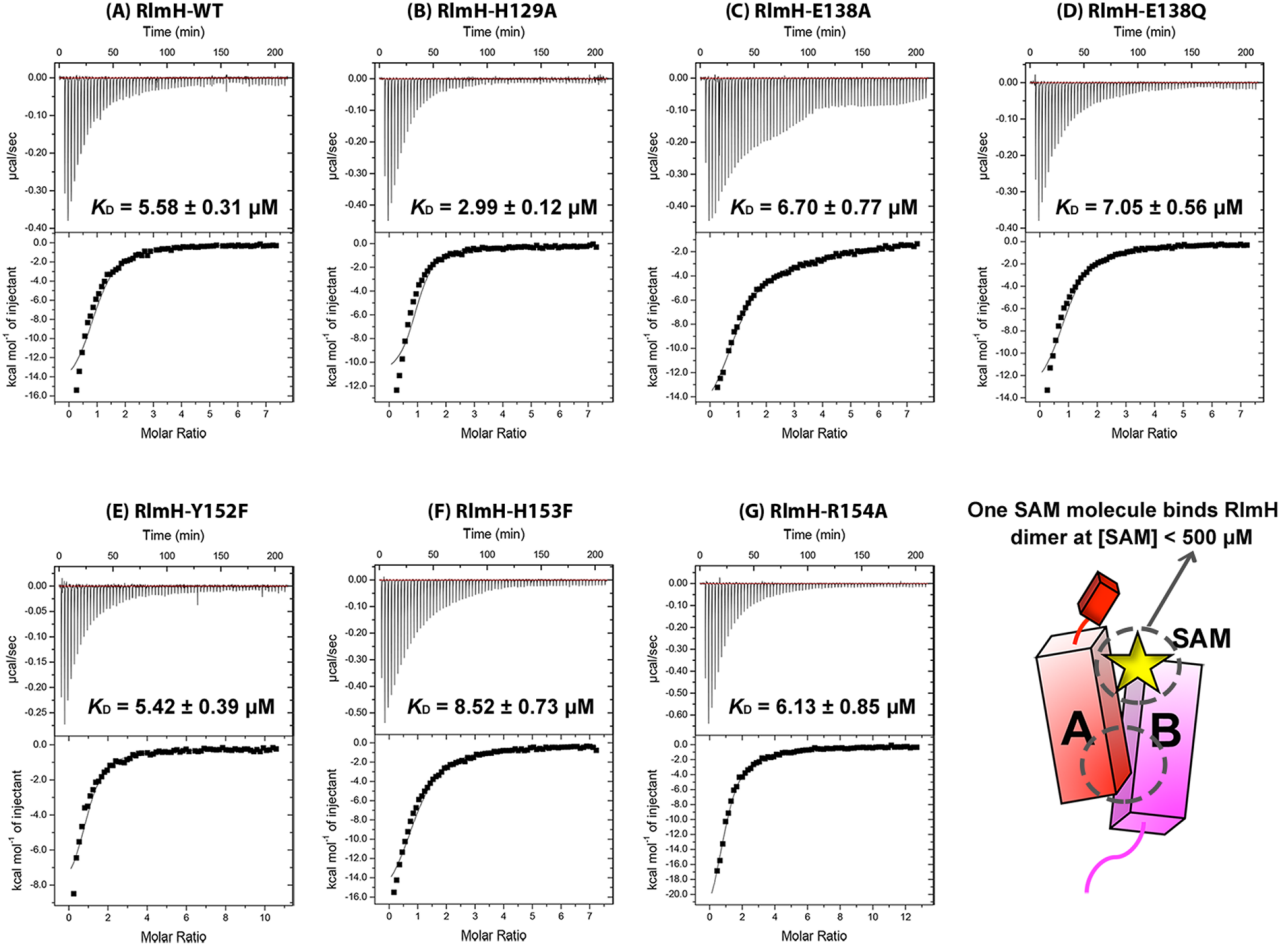
ITC analyses of SAM binding to wild-type or mutant RlmH. (**A**) 50 µM RlmH wild-type; **(B)** 51 µM RlmH-H129A; **(C)** 50 µM RlmH-E138A; **(D)** 51 µM RlmH-E138Q; **(E)** 35 µM RlmH-Y152F; **(F)** 51 µM RlmH-H153F and **(G)** 29 µM RlmH-R154A. Dissociation constants (*K*_*D*_) of SAM for each RlmH mutant are calculated from three replicates (except R154A, *n* = 2). Data were fitted using a sequential binding model. The c-value was 10. See also Figure S7.

We next used isothermal titration calorimetry (ITC) to test whether each RlmH mutant retained wild-type affinity for binding SAM (Figs 3 and S6). For the mutant proteins, the apparent equilibrium dissociation constants for the first SAM binding site in the RlmH dimer (*K*_D1_) ranged from 3.0 ± 0.1 (for H129A) to 8.5 ± 0.7 µM, similar to that of wild-type RlmH (5.6 ± 0.3 µM; Table 2). The modestly increased SAM binding of the H129A mutant is likely due to non-polar environment in this region bolstered by the adjacent proline residues. The enhanced binding, however, comes at the cost of modestly decreased catalytic activity.

**Table 2:** Isothermal titration calorimetry measurements of SAM binding affinity to wild-type and mutant RlmH.

Complex	Calculated *K*_D1_ (μM)	Δ*H* (kcal mol^−1^)	-TΔ*S* (kcal mol^−1^)	Δ*G* (kcal mol^−1^)
WT	5.58 ± 0.31	−15.48 ± 0.68	0.70 ± 0.14	−14.79 ± 1.48
H129A	2.99 ± 0.12	−10.53 ± 0.51	0.25 ± 0.15	−10.28 ± 1.61
E138A	6.70 ± 0.77	−17.18 ± 0.41	0.85 ± 0.13	−16.33 ± 1.23
E138Q	7.05 ± 0.56	−15.48 ± 0.54	0.71 ± 0.07	−14.77 ± 0.67
Y152F	5.42 ± 0.39	−9.47 ± 0.38	0.19 ± 0.11	−9.28 ± 1.05
H153F	8.52 ± 0.73	−19.53 ± 0.66	1.06 ± 0.17	−18.47 ± 1.73
R154A	6.13 ± 0.85	−29.45 ± 0.69	1.87 ± 0.14	−27.57 ± 1.52

The table reports standard deviations for three replicates (except for R154A mutant, n = 2).

The thermodynamic parameters are displayed in Figure S7.

*K*_D1_ of wild-type and mutant RlmH are similar to those of other SPOUT methyltransferases: 4.5 µM for *Sp*Trm10^55^; 7.6 µM for *Sc*Trm10^55^; 20.0 µM for *Hi*YibK/TrmL^56^; 33.0 µM for *Sa*Trm10^57^. The second binding site in the RlmH dimer remained unoccupied at SAM concentrations below 500 µM, consistent with the *K*_D2_ ~1 mM reported for OrfX^45^. A millimolar *K*_D2_ is consistent with our observation of two SAM molecules per dimer in the crystal structure, which was obtained using 2 mM SAM in the crystallization solution. None of the mutated residues directly contacts SAM in our structure (Fig. 1), consistent with the lack of effect of the mutations on SAM binding. We conclude that the loss of activity in mutant RlmH does not reflect a SAM-binding defect.

### R154A mutant is deficient in ribosome binding

**Figure 4:**
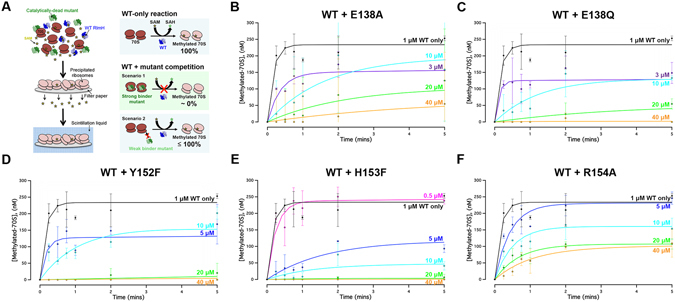
Competition analyses of RlmH mutants. (**A**) Scheme for the *in vitro* competitive methylation assay. (**B–F**) Time progress curves for methylation of Δ*rlmH*-70S ribosomes by wild-type RlmH (1 µM) in competition with E138A, E138Q, Y152F, H153F and R154A mutants at different concentrations. The same curve for methylation by wild-type RlmH is shown (black) in all panels for reference. Error bars show error of fit for two independent experiments.

To test whether the loss of catalytic activity in the RlmH mutants reflected a failure to bind ribosomes, we performed a competition assay in which the methylation efficiency of wild-type RlmH was measured in the presence of mutant proteins. If a mutant protein does not bind the ribosome, the efficiency of methylation by wild-type RlmH will be unaltered. In contrast, if a mutant protein retains near wild-type ribosome binding, competition of RlmH with the catalytically-inactive, mutant protein should reduce the efficiency of methylation. The competition assay was performed under single-turnover conditions, in which the concentration of wild-type RlmH was higher than the concentration of 70S ribosomes; varying concentrations of each catalytically inactive mutant were incubated with ribosomes before the addition of wild-type RlmH (Fig. 4).

Of the five inactive mutants, four inhibited wild-type RlmH. The two E138 mutants, Y152F, and H153F halved the extent of methylation at ~3 µM mutant concentration; with 20–40 µM mutant protein, no methylation by wild-type RlmH was detected. Thus, none of these four mutations impaired ribosome binding. At 5 µM, the R154A mutant had little effect on methylation by RlmH, and at 40 µM reduced methylation by just two-fold. Thus, mutation of R154 compromised ribosome binding. The presence of some ribosome binding at the higher concentration suggests that the R154A mutant is deficient in both ribosome binding and catalysis.

## Discussion

Our data suggest that *E. coli* RlmH functions as a dimer, with the SAM binding pocket of one monomer and the C-terminal residues of its counterpart forming a composite active site. We propose that the C-terminal tail provides a residue that acts as a general base (see discussion below), while the other conserved residues form a network of interactions that organize the catalytic site. Like other SPOUT methyltransferases^58^ RlmH forms a dimer at concentrations at which RlmH efficiently methylates the 70S ribosome (Figs 1 and 2). Despite differences in sequence, dimer topology and substrate specificity, the conserved overhand-knot architecture of SPOUT family members enables a dimer to position the SAM binding pocket of one monomer next to the catalytic residues of the second monomer. Such architecture of the composite active site appears to be a general feature of SPOUT methyltransferases.

**Figure 5:**
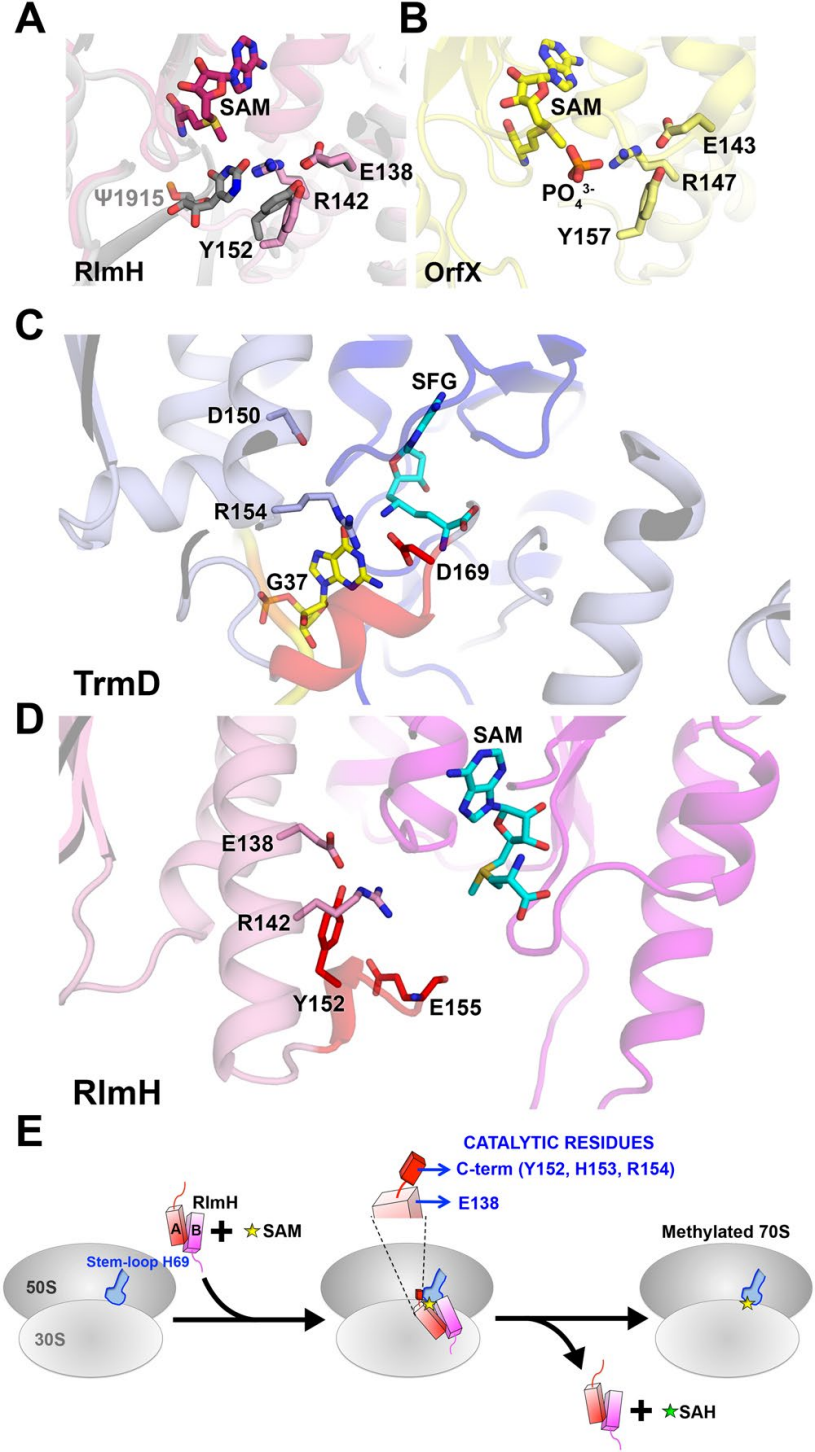
Comparison of the active sites of RlmH and other SPOUT methyltransferases (OrfX and TrmD), containing the conserved D/E-R-Y residues. (**A**) Predicted model of the 70S· RlmH · SAM complex (gray; Purta *et al*.^31^) and SAM-bound RlmH (magenta, this work). SAM is shown in sticks (magenta). Predicted position of Ψ1915 is shown with sticks. (**B**) The active site of *S. aureus* rRNA methyltransferase OrfX (yellow) in complex with SAM (yellow, sticks) and a phosphate ion (orange) (PDB ID 4FAK). SAM is bound to one OrfX monomer, while the residues shown in sticks are provided by the second monomer, as in *E. coli* RlmH. Conserved (Asp/Glu), Arg and Tyr interacting with the substrate nucleotide/analogs are shown in sticks and labeled. (**C**) The active site of *Haemophilus influenza* TrmD (light blue/dark blue) in complex with sinefungin (SFG, cyan, sticks) and tRNA (yellow) (PDB ID 4YVI). (**D**) The active site of *E. coli* RlmH (magenta/pink) in complex with SAM (cyan, sticks), shown in a view similar to that for the active site of *H. influenza* TrmD in **(C)**. The catalytic C-terminal tail is colored in red. (**E**) Schematic of the mechanism of Ψ1915 methylation by RlmH. See also Figures S5, S6 and S8.

Comparison of the SAM-bound RlmH structure with the crystal structure of apo-RlmH (PDB ID 1NS5) supports the earlier proposal^31, 45^ that the RlmH catalytic pocket, comprising the SAM-binding and Ψ1915-binding sites, lies at the interface of two monomers (Fig. 5). Our SAM-bound structure reveals that SAM binding shifts the C-terminus of RlmH by as much as 14 Å, rearranging it into a 3_10_ helix (Fig. 1, close-up). No other large rearrangements were observed. Kinetic analyses of RlmH mutants identify the conserved E138 and C-terminal residues Y152, H153 and R154 as essential for catalysis. The side chains of these amino acids lie ≥8 Å from the methyl group of SAM. Recent structures of a related tRNA-methyltransferase, TrmD, suggest that the proposed general base D169 can shift from ~7 Å to ~4.5 Å toward the methyl moiety of SAM upon tRNA binding^47^. Superposition of our RlmH structure with that of TrmD reveals that of the four catalytically critical residues in RlmH, Y152 is closest to D169 of TrmD (Figs 5 and S8). Loss of RlmH activity upon removal of the tyrosine hydroxyl group in the Y152F mutant is consistent with this residue serving as a general base, echoing the roles of catalytic tyrosines in some non-SPOUT methyltransferases^59, 60^. We propose that the interaction of Y152 with R142, which itself forms a salt bridge with E138, serves to perturb the *pK*_*a*_ of Y152, as would be required for this residue to act as a general base. Our mutational studies support a role for interactions within the E138-R142-Y152 triad in this process, as RlmH activity is substantially reduced in the R142 and E138 mutants. E138, R142 and Y152 are conserved widely among RlmH orthologs^28, 31, 37^.

H153 is also conserved, and an H153F mutant RlmH lacks detectable methyltransferase activity. The H153 side chain is rotated away from the methyl group of SAM and makes a hydrogen bond with the hydroxyl group of residue 145, which is always serine or threonine in RlmH orthologs. In apo-RlmH, the H153 side chain is oriented toward solvent and does not contact S145. Therefore, H153 likely stabilizes the formation of the C-terminal 3_10_-helix, which helps organize the RlmH active site. The penultimate residue of RlmH, R154, is replaced by lysine or histidine in some RlmH orthologs. In a small subset of RlmH orthologs, H153 is the terminal residue; these enzymes completely lack R154 and E155 (*E. coli* numbering). Our data suggest that R154 plays role in RNA binding or positioning: an R154A mutant was defective in ribosome binding and lacked catalytic activity even when its low affinity for ribosomes was compensated by using a high protein concentration (Fig. 4). In RlmH orthologs ending with H153 perhaps another residue adopts the role of R154.

The SPOUT methyltransferase superfamily contains proteins with diverse substrate specificities, primary structures, and lengths. RlmH comprises just 155 amino acids (~17 kDa), whereas Trm3p contains 1436 amino acids (~164 kDa). Yet all SPOUT methyltransferases form a common overhand knot structure. In addition to this overhand-knot architecture, all SPOUT methyltransferases use SAM as the methyl donor. It is therefore likely that the overhand knot architecture has been evolutionarily constrained by positive selection to retain the ability to bind SAM, particularly SAM in a bent conformation^58, 61^. Structural alignment of bacterial and archaeal SPOUT methyltransferase structures containing SAM or SAM-like ligands reveals that the three SAM pocket-forming bights of RlmH are also present in other methyltransferases, albeit with variable lengths and topologies (Figures S5 and S6). Bight 1, the least conserved among the three, contains a conserved threonine that interacts with the adenine moiety of SAM. Bight 2 contains a canonical glycine-rich motif (Figure S9), which is also found in Rossman-fold enzymes^62^. Bight 3 lies adjacent to helix α5 (in RlmH), which participates in both dimerization and catalysis by displaying E138 and R142 on the same side of the helix. A similar arrangement of glutamic or aspartic acid followed by an arginine or lysine a few residues away (Figures S5 and S6) exists in other bacterial SPOUT methyltransferases, consistent with the functional importance of this motif. The role of the general base, however, is likely played by different residues in SPOUT subfamilies. While larger methyltransferases employ an aspartate or glutamate, such as D169 in TrmD, RlmH lacks a similarly positioned conserved acidic side chain. Instead, a conserved tyrosine is positioned in the same area of the catalytic pocket (Figure S8), supporting its proposed role in catalysis. Closely positioned R142 and H129 may be involved in catalysis. Their mutations to alanine, however, only modestly reduce k_cat_, suggesting that the direct role is unlikely unless a structural reorganization or a water molecule compensates for the loss of the side chain function. It is also possible that the less conserved E155 or the C-terminal α carboxyl group of RlmH acts as the general base. The general-base role of the terminal α carboxyl group has been shown for some unrelated enzymes, such as phospholipase^63, 64^ and N-myristoyltransferase^65^.

The RlmH dimer allows formation of the SAM binding pocket within one monomer, while the second monomer provides its C-terminal tail to complete the catalytic site (Fig. 5). Monomer B also stabilizes the SAM binding pocket of monomer A: SAM-binding bights 1 and 3 from one monomer interact with the same bights of the second monomer *via* hydrophobic interactions that are conserved among RlmH orthologs (Figs 1 and S2). The mechanism of RlmH provides new insights into the evolution and conservation of the overhand-knot SPOUT methyltransferases dimers and small-protein catalysts in general. Our structures suggest that SAM binding “pre-arranges” the C-terminal tail for binding of the RNA substrate and catalysis. Structures of ribosome-bound RlmH are required to provide additional insights into the mechanism of substrate recognition and catalysis.

## Methods

### Construction and purification of recombinant wild-type and mutant RlmH

*rlmH* from *E. coli* JW0631 (ASKA Clone of the National Bio Resource Project, Keio University, Tokyo, Japan) was cloned into pET24b (Novagen, Darmstadt, Germany), using primers shown in Table S2. Gene sequencing confirmed proper subcloning of the *rlmH* gene, in which the first methionine is replaced by MHHHHHHV. The His_6_-tagged RlmH protein was expressed in BLR(DE3) cells (Novagen) at 37 °C. 1 mM isopropyl β-D-1-thiogalactopyranoside (IPTG) was added at OD_600_ = 0.8. Following overnight growth at 16 °C, cells were harvested, suspended in buffer A (50 mM Tris-HCl, pH 7.5, 300 mM KCl, 10 mM imidazole, 5 mM β-mercaptoethanol), lysed using a microfluidizer at 18,000 psi, and clarified by centrifugation at 38,397 × *g* for 20 min. This clarified lysate was incubated with 1 ml of nickel resin for 1 h at 4 °C. Using batch/gravity flow immobilized metal ion affinity chromatography (IMAC), lysate with the affinity matrix resin were then packed into a pierce centrifuge column. Following binding of the His_6_-tagged protein, the column was washed with buffer A. RlmH was eluted with buffer B (50 mM Tris-HCl, pH 7.5, 300 mM KCl, 250 mM imidazole, 5 mM β-mercaptoethanol). Eluted samples were buffer-exchanged into buffer A prior to the next purification step. Samples (in buffer A) were further purified by an additional IMAC purification step using a fast protein liquid chromatography system (GE Healthcare) and a 5 ml HisTrap column (GE Healthcare) with linear gradient elution up to 250 mM imidazole. Protein was further purified using size exclusion chromatography on a Superdex75 column (GE Healthcare). Gel filtration buffer contained 50 mM sodium diphosphate, pH 7.5, 300 mM KCl, 10% glycerol, 5 mM β-mercaptoethanol. Purified recombinant protein was concentrated using a 10-kDa cutoff centrifugal filter concentrator (Millipore). Prior to *in vitro* experiments, protein was dialyzed overnight in buffer C (50 mM sodium phosphate, pH 6.8, 200 mM KCl, 5% glycerol, 1 mM β-mercaptoethanol) to remove endogenously bound SAM or other ligands. Protein was stored at 4 °C.

Site-directed mutagenesis was performed using QuikChange II site-directed mutagenesis protocol (Stratagene, La Jolla, CA, USA). Seven mutants of RlmH were generated (H129A, E138Q, E138A, R142A, Y152F, H153F and R154A) by PCR using plasmid pET24b-RlmH as a template in combination with mutagenic primers (Table S2). Mutations were confirmed by DNA sequencing. Mutant proteins were expressed and purified as the wild-type protein.

### Size exclusion chromatography coupled with static laser light scattering

Dialyzed recombinant proteins were buffer-exchanged into the size-exclusion chromatography/multi-angle light scattering (SEC/MALS) running buffer (50 mM potassium phosphate, pH 7.0, 300 mM KCl, 2 mM DTT and 5% glycerol). Two amounts (0.35 mg/ml in 50 µl and 3.85 mg/ml in 550 µl) of each protein sample were subjected independently to SEC/MALS analysis. Each filtered protein sample was subjected to a Superdex S-200 HR10/30 column (GE Healthcare) connected to an Agilent 1200 High Performance Liquid Chromatography System equipped with an auto sampler (Agilent Technologies, Wilmington, DE, USA). The eluate from SEC was monitored by a photodiode array (PDA) UV/VIS detector, UV, (Agilent Technologies), Optilab rEX differential refractometer, (Wyatt Technology, Santa Barbara, CA, USA), static and dynamic, HELEOS II multi-angle laser light scattering detector with QELS capability (Wyatt Technology). The system was equilibrated in SEC/MALS running buffer (50 mM potassium phosphate, pH 7.0, 300 mM KCl, 2 mM DTT and 5% glycerol) at a flow rate of 0.5 ml/min. Weighted average molecular weights were determined across the entire elution profile using 1 sec intervals as previously described^66^. Data were collected and analyzed with the ASTRA software (version 5.3.4.20) at the Keck Biophysics Resource Facility at Yale University.

### Isothermal Titration Calorimetry (ITC)

ITC experiments were performed in 50 mM sodium phosphate, pH 6.8, 200 mM KCl, 5% glycerol, 1 mM β-mercaptoethanol (buffer C) with dialyzed RlmH protein (50 µM in the cell chamber) in the presence of SAM. We did not observe background buffer protonation or interaction between the buffer and RlmH or SAM. ITC experiments were conducted in an iTC_200_ system (MicroCal, Northampton, MA, USA) using one 0.4 µl injection followed by 69 0.5 µl injections of 1 mM SAM into 200 µl RlmH in the sample cell with constant stirring (1,000 rpm) at 25 °C. The intervals between SAM injections were 180 sec. The reference power was 10 µcal/sec; the cell concentration assumed that RlmH was homodimeric in the solution. Data were integrated using MicroCal Origin7 (MicroCal) correcting for ligand heats of dilution. Errors (S.D.) correspond to best curve fitting errors. A model of sequential binding sites was used to fit the experimental data.

### Δ*rlmH E. coli* 70S ribosome preparation

*ΔrlmH E. coli* (strain JW0631-1, Coli Genetic Stock Center, Yale, New Haven, CT, USA) were grown at 37 °C in LB medium to OD_600_ = 0.8 and collected by centrifuging at 4,000 × *g* for 20 min. The resulting cell pellet was resuspended in 30 ml of buffer A (20 mM Tris-HCl, pH 7.0, 10.5 mM MgCl_2_, 100 mM NH_4_Cl, 0.5 mM EDTA, 6 mM β-mercaptoethanol). Cells were lysed in a microfluidizer (Microfluidics, Westwood, MA, USA) at 18,000 psi and clarified by centrifugation at 38,397 × *g* for 20 min at 4 °C. The resulting supernatant was centrifuged again to yield the clarified lysate. The lysate was layered onto an equal volume of 1.1 M (37.7%) sucrose cushion in buffer B (20 mM Tris-HCl, pH 7.0, 15 mM MgCl_2_, 500 mM NH_4_Cl, 0.5 mM EDTA, 6 mM β-mercaptoethanol), and centrifuged at 214,573 × *g* for 16 h in a Ti45 rotor (Beckman Coulter, Danvers, MA, USA). Ribosome pellets were drained and quickly rinsed with 5 ml buffer C (20 mM HEPES · KOH pH 7.0, 6 mM magnesium acetate, 30 mM NH_4_Cl, and 6 mM β-mercaptoethanol). Each pellet was then resuspended in 5 ml of buffer C, transferred to 1.5 ml tubes, and clarified by centrifuging at 17,000 × *g* for 5 min. The supernatant was diluted with 40 ml buffer B, and the final concentration of NH_4_Cl was adjusted to 500 mM. This solution was loaded into Ti70 tubes and topped up with buffer B, centrifuged at 351,046 × *g* for 2 h in a Beckman Ti70 rotor. The pellet was resuspended in 0.5 ml of buffer C and layered onto a 10–35% (w/w) sucrose gradient in buffer C, then centrifuged at 71,999 × *g* for 13 h in a SW-28 rotor (Beckman Coulter). Gradients were analyzed by continuous monitoring of the absorbance at 254 nm. The 70S ribosome peak (~5 ml) was collected and buffer exchanged to buffer C using a 100-kDa cutoff centrifugal filter concentrator (Millipore, Billerica, MA, USA). The resulting partially purified ribosomes were subjected to a second round of 10–35% (w/w) sucrose gradient purification to remove contaminating 50S ribosomes. The purity of the *ΔrlmH* 70S ribosomes was verified by analytical sucrose gradient (centrifugation at 273,865 × *g* for 2 h in a Beckman SW-41 rotor). Purified ribosomes in buffer C were flash frozen in liquid nitrogen and stored as small aliquots at −80 °C.

### *In vitro* methylation assays

*ΔrlmH* 70S ribosomes were heat-activated for 5 min at 42 °C and then gradually cooled to 25 °C. RlmH protein was pre-incubated with [^3^H]-*S*-adenosyl-L-methionine (Perkin Elmer, Waltham, MA, USA) at 25 °C for 30 min. We monitored methylation using 50 µM [^3^H]-SAM. The SAM concentration was >*K*_*M*_ for SAM^33^, consistent with the occupancy of one binding site per RlmH monomer. Testing whether occupancy of the second SAM binding site affects the rate of methylation was not feasible in the current assay.

Methyltransferase activity was measured using 0.3 µM *ΔrlmH E. coli* 70S ribosomes, 0.1–40 µM purified RlmH protein (protein concentration varied in titration experiments) and 50 µM [^3^H]-*S*-adenosyl-L-methionine in methylation buffer (50 mM Tris/HCl at pH 7.0, 100 mM NH_4_Cl, 10 mM MgCl_2_, 1 mM DTT, 0.01% Igepal) at 25 °C. At each time point, 5 µl of the reaction were quenched with 1 ml of ice-cold 10% trichloroacetic acid (TCA). Quenched samples were then incubated for 30 min on ice. The precipitate was collected on Whatman GF/C glass fiber filters (Whatman, Marlborough, MA, USA) pre-wetted with ice-cold 10% TCA (30 min prior to experiments), washed with 3 ml 10% cold TCA, 2 ml ice-cold 70% ethanol and dried for 30 min. The amount of [^3^H]-labeled ribosomes on the filter paper was quantified a Tri-Carb scintillation counter (Perkin Elmer, Waltham, MA, USA) using 5 ml of biodegradable liquid scintillation cocktail (Atlantic Nuclear Corp, Rockland, MA, USA).

### Substrate binding competition assay

Substrate binding competition assays were performed as described for the methylation assay except that a range of concentrations of mutant protein was incubated with activated ribosomes for 20 min at 25 °C before wild-type RlmH · [^3^H]-SAM was added.

### Rate analyses

The rate of methyl group incorporation into *ΔrlmH E. coli* 70S ribosomes was calculated by fitting to y = y_0_ + A(1 − e^−kt^), where dy/dt = Ake^−kt^. The initial velocity dy/dt_0_ = Ak^67^. Michaelis-Menten kinetics were determined using GraphPad Prism 6.0 g (GraphPad Software, San Diego CA USA) and values are reported in Table 1. Since the fitted K_M_ for RlmH is lower than the concentration of the substrate (≤0.3 uM), the <or> symbols are used to reflect the uncertainties in k_cat_/K_M_ or Fold Changes. Burst kinetics data from the substrate binding competition assays were fit using Igor Pro 6.11 (WaveMetrics, Inc., Portland, OR, USA).

### Protein crystallization and data collection

Purified RlmH was concentrated to 10 mg/ml in the buffer containing 50 mM sodium diphosphate, pH 7.5, 300 mM KCl, 10% glycerol, 5 mM β-mercaptoethanol. Initial crystallization hits were obtained using Crystal Screens 1–2 (Hampton Research, Aliso Viejo, CA, USA) and Morpheus Screen (Molecular Dimensions Ltd., Newmarket, Suffolk, United Kingdom) using sitting-drop vapor diffusion in 96-wells plates. SAM- and sinefungin-bound RlmH crystals were obtained with Morpheus screen solution 21 containing 0.1 M Tris-bicine buffer (pH 8.5), 0.09 M NaF, 0.09 M NaBr, 0.09 M NaI, 20% v/v PEG 550 MME, 10% w/v PEG 20,000 in the presence of 2 mM SAM or sinefungin. With this initial hit condition, different protein to crystallization solution ratios were screened in 24-well plates. Larger crystals suitable for data collection were obtained in 24-well plates, *via* hanging-drop vapor diffusion, after 1 week at 16 °C. Drops were formed by mixing 2 µl protein and 2 µl crystallization solutions. Crystals were cryo-protected with 20% glycerol in reservoir solution and flash-cooled in liquid nitrogen.

Crystals were screened at beam line 24-ID-B and diffraction data were collected at beam line 24-ID-C at the Advanced Photon Source (APS) of Argonne National Laboratory (Argonne, IL, USA). Data were processed and integrated using XDS^68^, scaled with SCALA^69^ or AIMLESS^70^ in CCP4^71^. Crystals belonged to space group P2_1_ and the asymmetric unit contained 4 molecules (2 dimers).

### Structure determination and refinement

- Structures were solved by molecular replacement using MOLREP^72^ in CCP4^71^ and PHASER^73, 74^ in PHENIX^75^ using the apo *E. coli* RlmH crystal structure (PDB ID 1NS5) as the starting search model. Structural models were visualized and remodeled with COOT^76^. Refinement was performed using REFMAC5^77^ and PHENIX 1.9_1692^75^. Individual B-factors were refined together with each chain as TLS group. Final models were evaluated using the comprehensive validation option in PHENIX^75^ and yield good crystallographic and stereochemical statistics^78^ (Table 3). Figures were generated using PyMOL 1.3 (Schrödinger, Cambridge, MA, USA).

**Table 3:** Data collection, processing and refinement statistics. Statistics for the highest-resolution shell are shown in parentheses.

	RlmH · SAM	RlmH · SFG
Diffraction source	23-ID-C, APS	23-ID-C, APS
Detector	Dectris Pilatus 6M-F	Dectris Pilatus 6M-F
Wavelength (Å)	0.9792	0.9792
Unit-cell parameters (Å)	a = 66.53,	a = 66.37,
	b = 37.36,	b = 37.95,
	c = 122.07	c = 121.82
	α = *γ* = 90.00°,	α = *γ* = 90.00°,
	*β* = 104.99°	*β* = 105.74°
Space group	*P* 1 2_1_ 1	*P* 1 2_1_ 1
Resolution range (Å)	29.5–2.30	39.1–2.10
	(2.38–2.30)	(2.18–2.10)
Total No. of reflections	129594 (13342)	194310 (19231)
No. of unique reflections	26034 (2583)	34496 (3405)
Average multiplicity	5.5 (5.2)	5.6 (5.6)
Mean *I*/σ(*I*)	14.46 (2.18)	17.66 (1.95)
Completeness (%)	98.59 (98.47)	99.24 (93.52)
*R*_merge_^a^	0.0770 (0.755)	0.06791 (0.886)
*R*_meas_	0.0863	0.0749
CC_1/2_^b^	0.999 (0.742)	0.999 (0.679)
CC*	1.000 (0.923)	1.000 (0.899)
Wilson *B* factor (Å²)	43.16	36.84
Refinement with PHENIX		
*R*_work_	0.1771	0.1833
	(0.2369)	(0.2608)
*R*_free_^c^	0.2287	0.2380
	(0.3019)	(0.3199)
No of non-hydrogen atoms	5162	5278
No of protein atoms	5068	5068
No of ligands	4	4
No of waters	94	210
Protein residues	628	628
R.m.s.d.,^d^ bond lengths (Å)	0.009	0.013
R.m.s.d.,^d^ bond angles (°)	1.32	1.27
Average *B* factor (Å²)	63.50	53.30
Protein	63.70	53.30
Solvent	50.80	52.30
*MolProbity* Statistics		
Ramachandran, favored (%)	98.5	98.9
Ramachandran, allowed (%)	1.5	1.1
Ramachandran, outliers (%)	0	0
*MolProbity* rotamer outliers (%)	0.6	0
*MolProbity* clashscore	5.09	6.17
*MolProbity* score^e^	1.27	1.34
PDB ID	5TWJ	5TWK

^a^ *R*_merge_ and *R*_meas_ from PHENIX are reported^75^.

^b^CC_1/2_ is the correlation coefficient of the mean intensities between two random half-datasets^83^.

CC* is an estimate of the “true” CC of the data under examination to the (unknown) true intensities^83^.

^c^5% of the reflections were selected for free *R* calculation.

^d^Root-mean-square deviation from ideal values^84^.

^e^MolProbity score was calculated as defined in ref. 78.

### Sequence and structural analyses

- NCBI (PSI) BLAST^79^ was used to obtain 5,000 non-redundant RlmH homolog sequences. MUSCLE^80, 81^ was used to generate a multiple sequence alignment which was presented with WebLogo 3^82^.

## Electronic supplementary material

Supplementary Information
