# Supplementary material for: Small methyltransferase RlmH assembles a composite active site to methylate a ribosomal pseudouridine
Source: Sci Rep. 2017 Apr 20;7:969. doi: 10.1038/s41598-017-01186-5 (PMC5430550; doi:10.1038/s41598-017-01186-5)
Supplement: Supplementary file 1 — Supplementary Information [file 41598_2017_1186_MOESM1_ESM.doc]

**Small methyltransferase RlmH assembles a composite active site to methylate a ribosomal pseudouridine**

**Cha San Koh^1^, Rohini Madireddy^1^, Timothy J. Beane^1^, Phillip D. Zamore^1,2^,* and Andrei A. Korostelev^1^,***

^1^RNA Therapeutics Institute, University of Massachusetts Medical School, 368 Plantation St., Worcester, MA 01605, USA

^2^Howard Hughes Medical Institute

* Correspondence: [phillip.zamore@umassmed.edu](mailto:phillip.zamore@umassmed.edu); andrei.korostelev@umassmed.edu

**Supplementary Information:**

**Supplementary Figures S1-S9**

**Supplementary Tables S1, S2**

**Supplementary References**

**
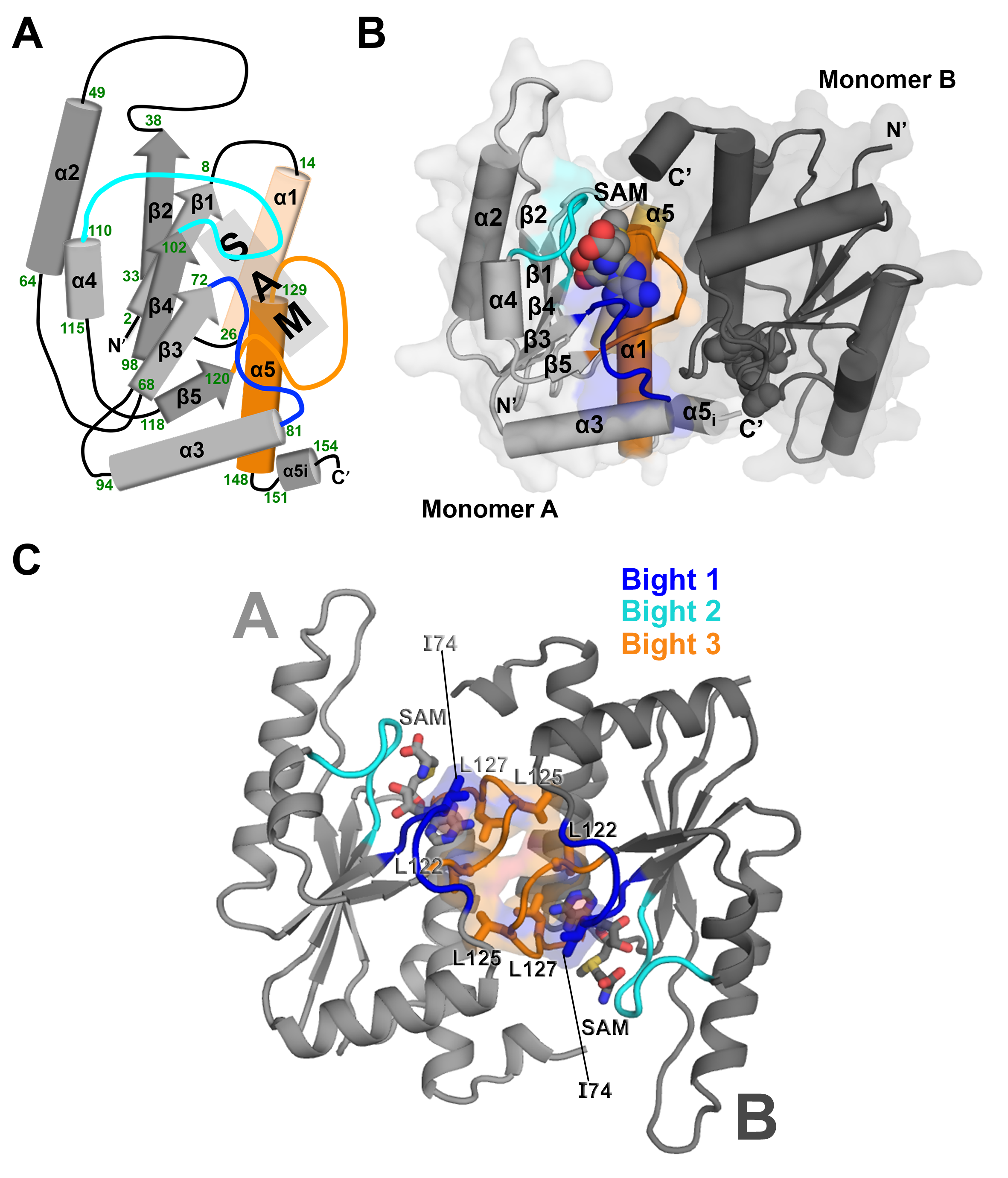
**

**Supplementary Figure S1. The knotted SPOUT-fold topology of RlmH.**

**(A)** Topology diagram of RlmH. α-helices are shown as gray cylinders; β-strands are shown as gray arrows. Dimerization-interface helices α1 and α5 are shown in light orange and orange, respectively. Three bights (bight 1: blue; bight 2: cyan; bight 3: orange) form the SAM binding pocket. The intertwining of bights 1 and 3 defines the overhand-knot topology of RlmH. Helices, beta strands and the connecting loops are numbered. The N and C termini are labeled. **(B)** Cartoon and surface representation of the RlmH dimer bound with SAM shown in ball-and-stick representation. Secondary-structure elements, bights and dimerization helices are labeled and colored as in (A). **(C)** Interactions between RlmH molecules in the dimer. Conserved hydrophobic residues in bights 1 and 3 are labeled and shown as sticks and semi-transparent surface.

**
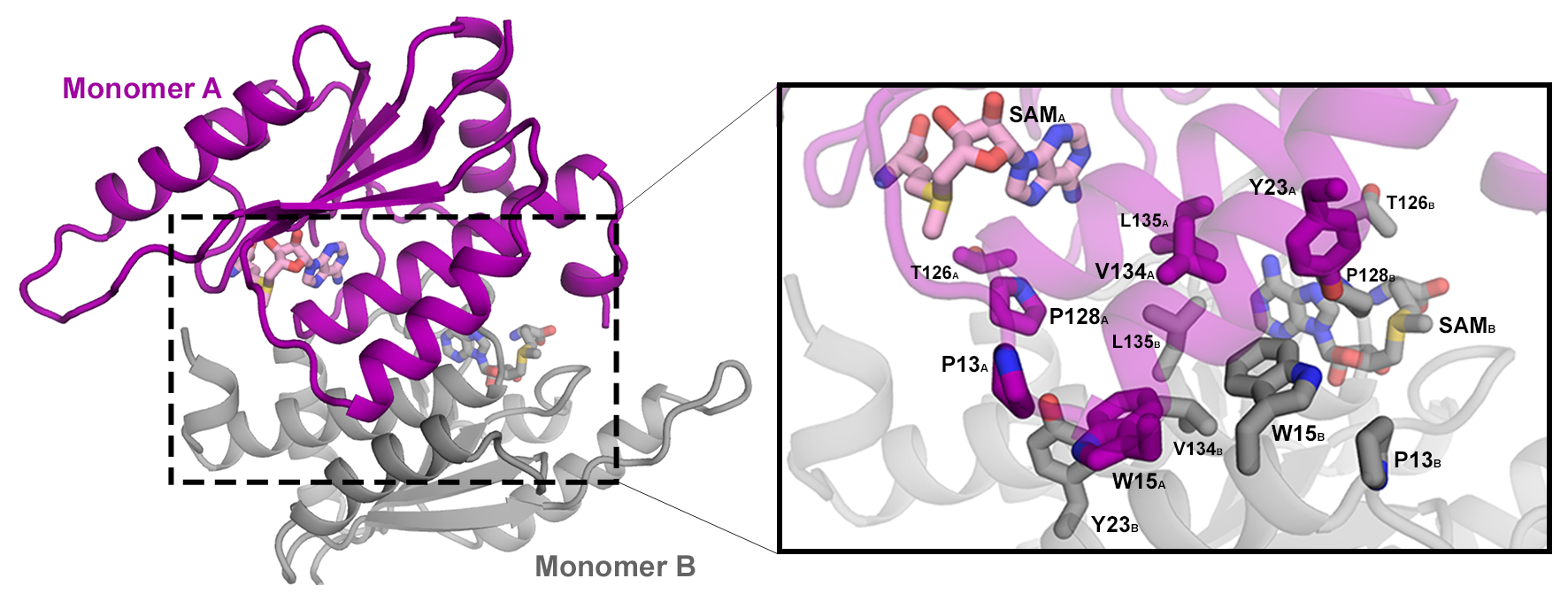
**

**Supplementary Figure S2. Dimerization interface of RlmH.**

RlmH•SAM dimer interface is mediated by two pairs of antiparallel helices (α1 and α5), which form a four-helix bundle. Conserved hydrophobic residues at the dimer interface are shown as sticks. Monomers are labeled and colored in magenta and gray.

**
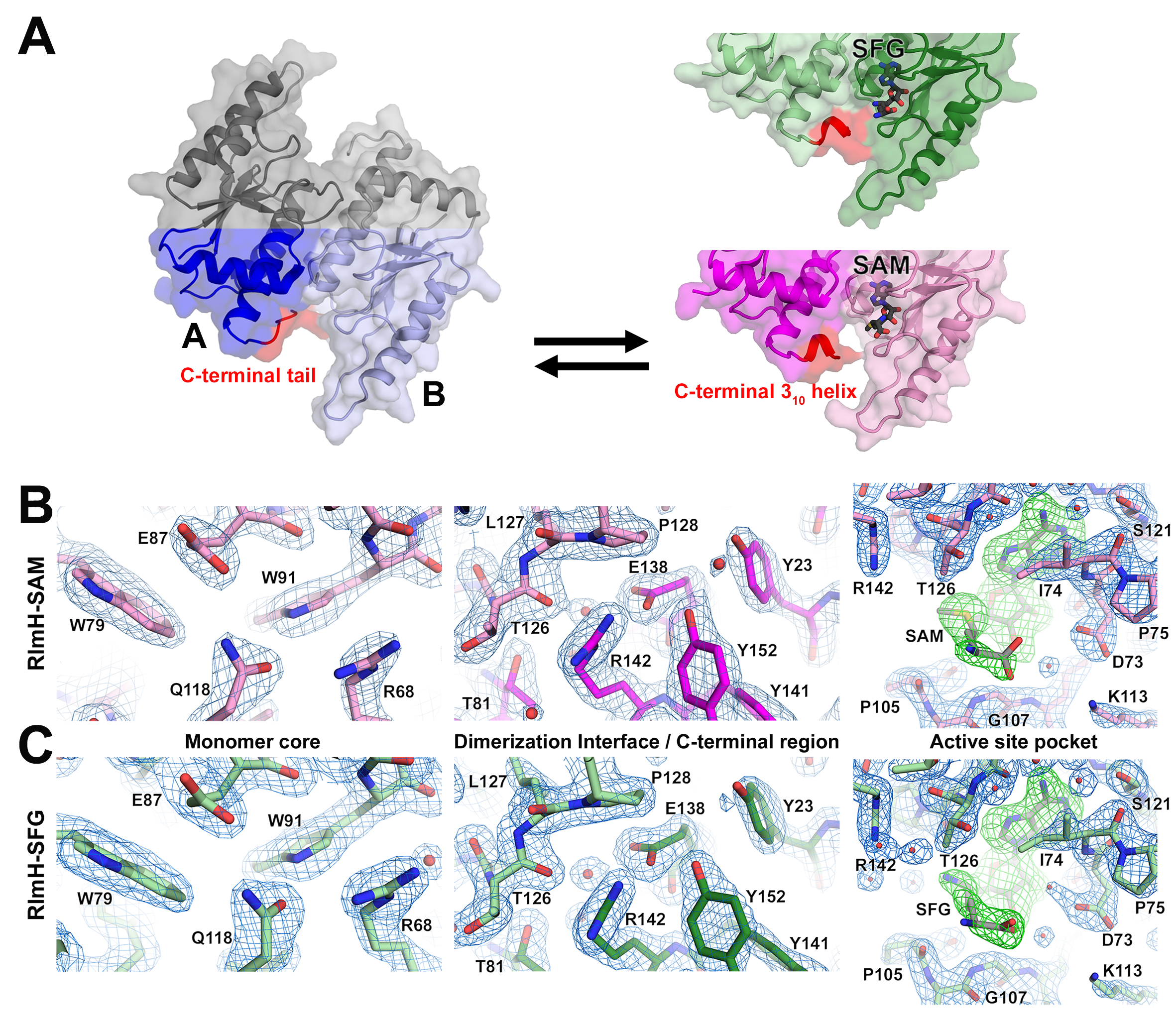
**

**Supplementary Figure S3. Rearrangements of the C-terminal tail upon binding of SAM or sinefungin to RlmH.**

**(A)** Positions of the C-terminal tail in ligand-free RlmH structure (blue/light blue, PDB ID 1NS5), RlmH•sinefungin structure (light green/green; this work) and RlmH•SAM structure (magenta/pink; this work). The C-terminal tail is shown in red. 2F_obs_-F_calc_ electron densities (blue mesh, contour level 2.5σ; calculated using PHENIX ^1^) for SAM and sinefungin are shown in boxes. **(B** and **C)** 2F_obs_-F_calc_ (blue) and F_obs_-F_calc_ (green) electron densities at the SAM binding site, at the monomer core and at the dimerization interface including the C-terminal region (contour level 2.5σ) are shown for the SAM-bound **(B)** and sinefungin-bound **(C)** RlmH (this work). Colors are as in **(A)**.

**
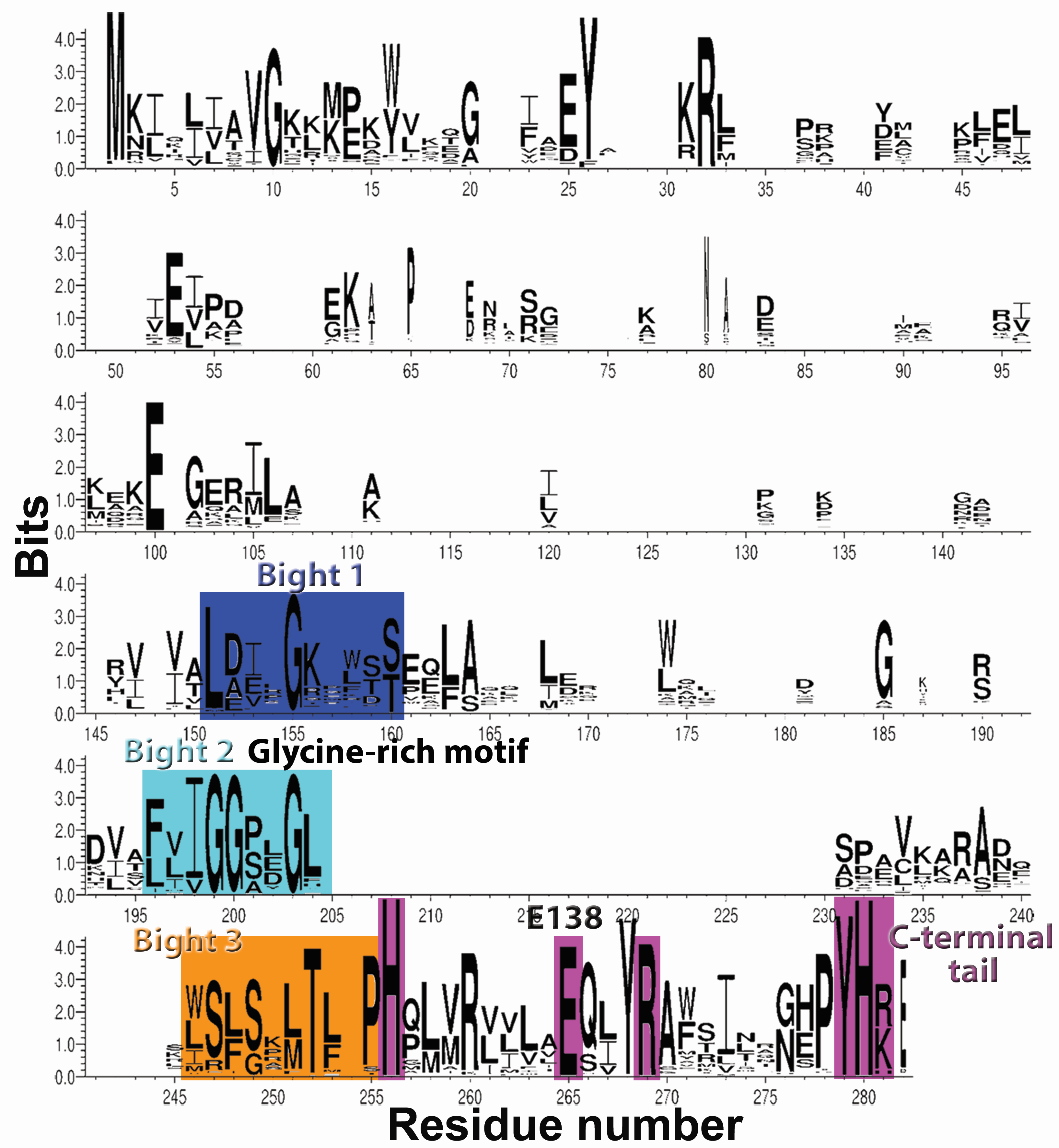
**

**Supplementary Figure S4. Sequence conservation among 5,000 non-redundant bacterial RlmH orthologs.**

The first residue for *E. coli* RlmH starts at methionine 43 in this representation. Residues mutated for kinetic analyses are highlighted in magenta. Bight 1 is highlighted in blue, bight 2 in cyan and bight 3 in orange.

**
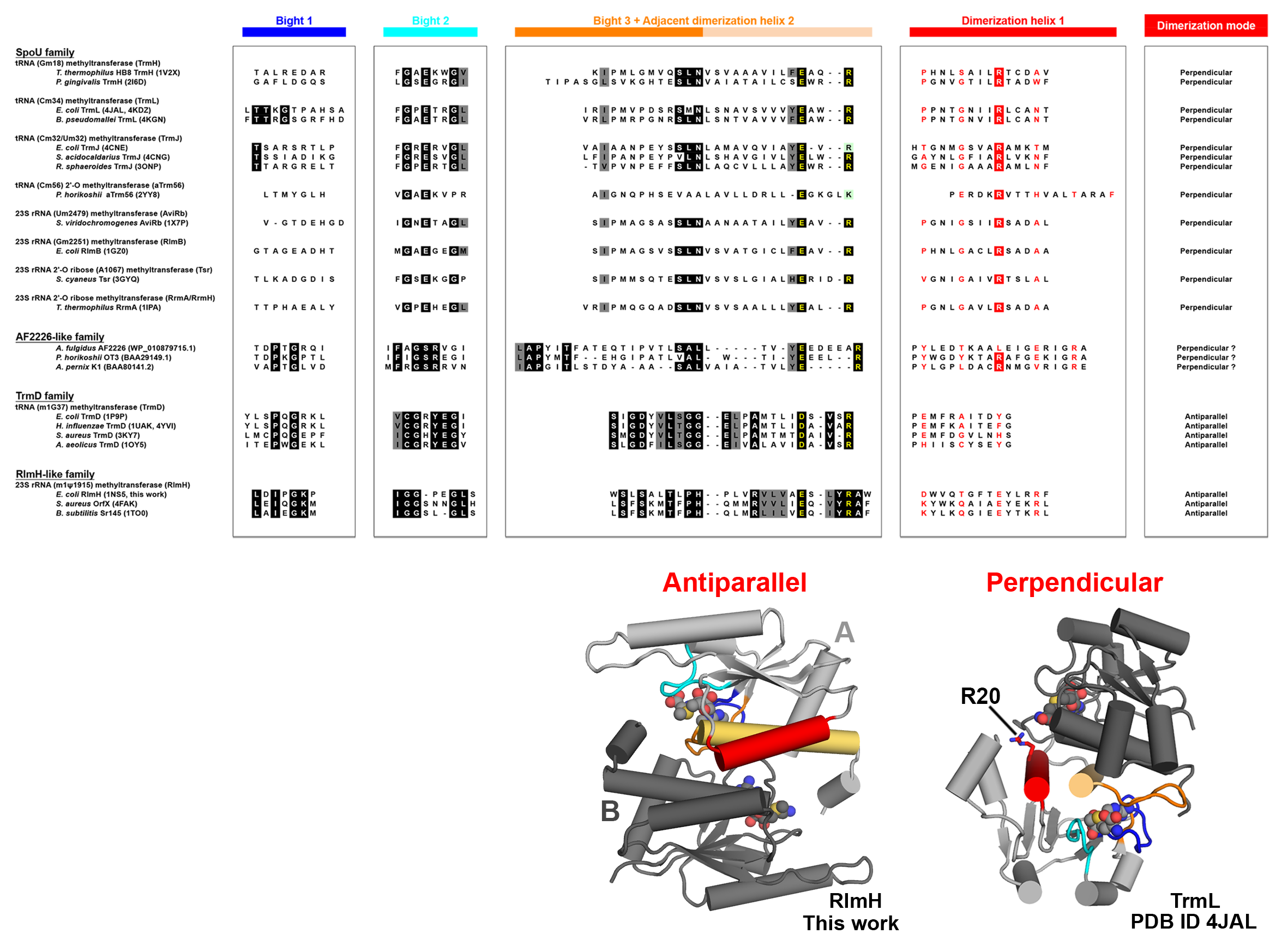
**

**Supplementary Figure S5. Comparison of dimer architectures of RlmH and larger SPOUT methyltransferases.**

The table shows amino acids that are involved in formation of similar structural features (shown in different colors) in various methyltransferases, which form antiparallel or perpendicular dimers (shown in lower panels). Structure-guided sequence alignment of bacterial and archaeal SPOUT methyltransferases was performed using crystal structures and reported sequence alignment ^2^.

**
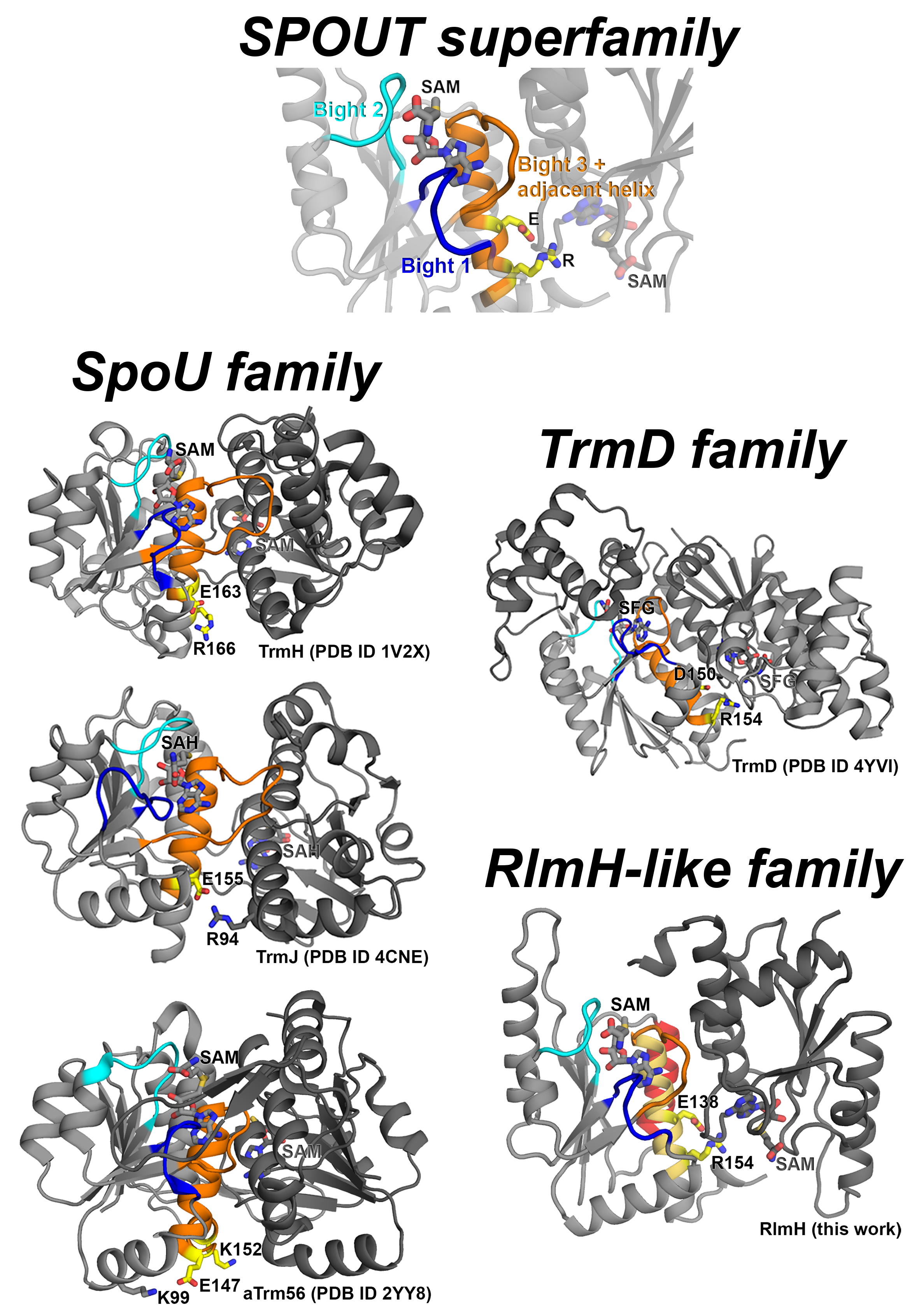
**

**Supplementary Figure S6. SAM-binding pocket is formed by the same structural motifs in SPOUT methyltransferases.**

The SAM-binding pocket of RlmH (upper panel) is formed by bights 1, 2 and 3, which are also present in larger SPOUT methyltransferases belonging to the SpoU and TrmD families. Bight 1 is shown in blue, bight 2 in cyan and bight 3 in orange. The adjacent helix 2, which is involved in the dimerization interface, is shown in orange. Conserved residues of helix 2 are shown as sticks.

**
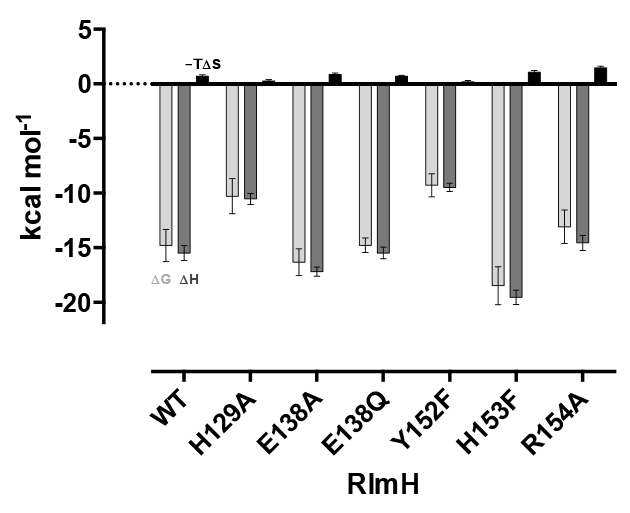
**

**Supplementary Figure S7. Thermodynamic parameters derived from ITC for SAM binding to RlmH.**

The thermodynamic parameters (ΔG, ΔH, -TΔS) for binding of the first SAM molecule to RlmH and RlmH mutants were obtained from isothermal titration calorimetry measurements (n=3; except R154A, n=2).

**
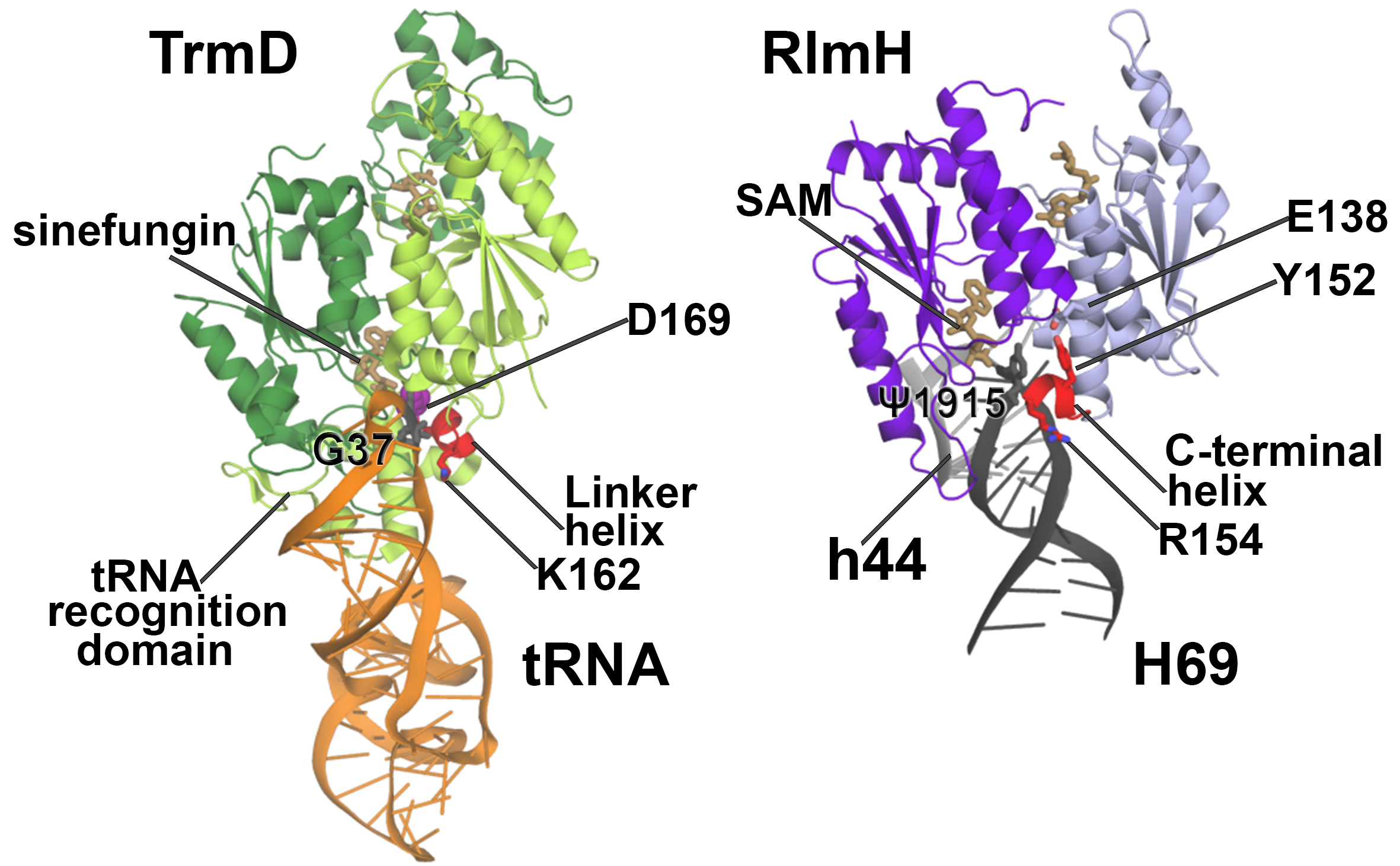
**

**Supplementary Figure S8. Comparison of the crystal structure of the tRNA**•**TrmD complex with the structural model predicted for the 70S**•**RlmH complex.**

Crystal structure of *Haemophilus influenzae* TrmD bound with sinefungin and tRNA (PDB ID 4YVI; ^3^ (left panel) and a model of the RlmH•SAM crystal structure (this work) docked to the *E. coli* 70S ribosome (right panel). The structural model is based on the structural model for *E. coli* RlmH docked into *T. thermophilus* 70S ribosome ^4^. Only parts of helices 44 (16S rRNA) and 69 (23S rRNA) of the 70S ribosome are shown for clarity. Two monomers of TrmD are colored in light and dark green; two monomers of RlmH are colored in light and dark blue. tRNA and helix 69 (H69) are colored in orange and dark gray respectively; helix 44 (h44) is colored in light gray; sinefungin and SAM are shown in wheat; the linker helix and C-terminal helix are in red; target nucleotides G37 and Ψ1915 are shown in gray sticks. Key catalytic residues are shown in purple (D169 in TrmD), red (Y152 and R154 in RlmH; K162 in TrmD) and light blue (E138 in RlmH). E138 of RlmH is also shown in sticks (light purple). The C-terminal tRNA-recognition domain in TrmD stabilizes the tRNA anticodon stemloop. RlmH lacks the C-terminal domain. h44 of the 30S subunit is positioned similarly to the C-terminal tRNA-binding domain with respect to the RNA substrate (H69), in keeping with the activity of RlmH on the 70S ribosome but not on the isolated 50S subunit ^5^.

**
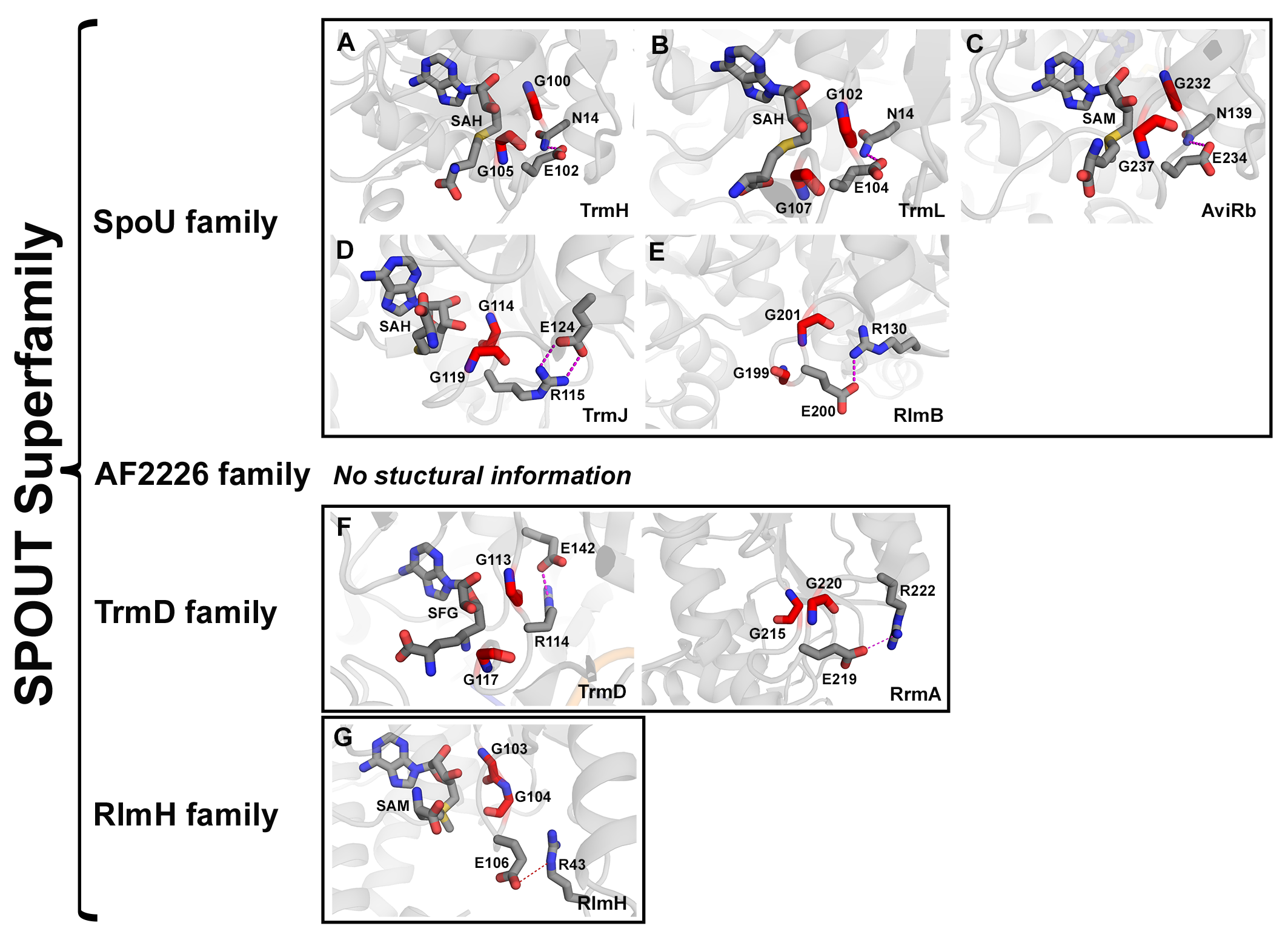
**

**Supplementary Figure S9. The glycine-rich loop and salt bridge near the SAM-binding pocket of RlmH and other SPOUT methyltransferases. (A-H)** SAM, glycine residues and salt-bridge-forming residues are shown as sticks. These structural features of RlmH (panel H; this work) are similar to those found in SpoU enzymes (A-F; PDB ID: 4KGN, 4JAL, 1X7P, 4CNE, 1GZ0, 1IPA), TrmD-like enzymes (G; PDB ID 4YVI).

| **Supplementary Table S1. SEC/MALS results** | | | | |
| --- | --- | --- | --- | --- |
| RlmH protein | Peak  Elution at UV trace  (ml) | MW^a^  Average for the eluting peak  (kDa) | MW Range observed across the eluting peak  (kDa) | Sequence-based MW for RlmH monomer  (kDa) |
| Wild-type | 16.9 | 37.2 | 37.0 – 37.5 | 18.3 |
| E138A | 16.8 | 37.8 | 37.3 – 38.5 | 18.2 |
| Y152F | 16.8 | 36.6 | 36.0 – 36.8 | 18.2 |
| H153F | 16.9 | 37.2 | 37.1 – 37.3 | 18.3 |
| R154A | 16.9 | 37.5 | 37.0 – 37.8 | 18.2 |
| ^a^. Molecular weight | | | | |

| **Supplementary Table S2. Primer pairs for site-directed mutagenesis used to obtain RlmH wildtype and mutants.** | | |  |
| --- | --- | --- | --- |
| **RlmH** | **Forward Primer Sequence** | **Reverse Primer Sequence** | |
| **WT** | CCCGCATATGCATCACCATCACCATCACGTGAAGCTGCAACTTGTCG | CCCGGGATCCTCACTCACGGTGATAAGGATGG | |
| **H129A** | CTTACCCTCCCGGCGCCGCTGGTTCGTGTTCTGGTT | AACCAGAACACGAACCAGCGGCGCCGGGAGGGTAAG | |
| **E138A** | CCGGTACAGACTCGCTGCGACCAGCAC | GTGCTGGTCGCAGCGAGTCTGTACCGG | |
| **E138Q** | GGTACAGACTCTGTGCGACCAGCACGC | GCGTGCTGGTCGCACAGAGTCTGTACC | |
| **R142A** | CTGGTTGCGGAATCTCTGTACGCGGCGTGGTCTATC | GATAGACCACGCCGCGTACAGAGATTCCGCAACCAG | |
| **Y152F** | CAACCATCCTTTTCACCGTGAGT | ACTCACGGTGAAAAGGATGGTTG | |
| **H153F** | CCATCCTTATTTTCGTGAGTGAGG | CCTCACTCACGAAAATAAGGATGG | |
| **R154A** | GAGCATCACCACCAACCATCCTTATCACGCGGAATGAGGTTCTGAA | TTCAGAACCTCATTCCGCGTGATAAGGATGGTTGGTGGTGATGCTC | |
| All sequences are shown in the 5’ – 3’ direction. Trinucleotides coding for the replacement amino acids are underlined. | | |  |

**Supplementary References:**

1 Adams, P. D. *et al.* PHENIX: a comprehensive Python-based system for macromolecular structure solution. *Acta Crystallogr D Biol Crystallogr* **66**, 213-221, doi:10.1107/S0907444909052925 (2010).

2 Anantharaman, V., Koonin, E. V. & Aravind, L. SPOUT: a class of methyltransferases that includes spoU and trmD RNA methylase superfamilies, and novel superfamilies of predicted prokaryotic RNA methylases. *J Mol Microbiol Biotechnol* **4**, 71-75 (2002).

3 Ito, T. *et al.* Structural basis for methyl-donor-dependent and sequence-specific binding to tRNA substrates by knotted methyltransferase TrmD. *Proc Natl Acad Sci U S A* **112**, E4197-4205, doi:10.1073/pnas.1422981112 (2015).

4 Purta, E., Kaminska, K. H., Kasprzak, J. M., Bujnicki, J. M. & Douthwaite, S. YbeA is the m3Psi methyltransferase RlmH that targets nucleotide 1915 in 23S rRNA. *RNA* **14**, 2234-2244, doi:10.1261/rna.1198108 (2008).

5 Ero, R., Peil, L., Liiv, A. & Remme, J. Identification of pseudouridine methyltransferase in Escherichia coli. *RNA* **14**, 2223-2233, doi:10.1261/rna.1186608 (2008).
